# IMPACT OF A NURSING INTERVENTION ON PHYSICAL ACTIVITY AND INDEPENDENCE IN NURSING HOME RESIDENTS

**DOI:** 10.1093/geroni/igae098.1951

**Published:** 2024-12-31

**Authors:** Michel Bleijlevens, Mirre den Ouden, Judith Meijers, Sandra Zwakhalen, Erica De Vries, Jan Hamers

**Affiliations:** Maastricht University, Maastricht, Limburg, Netherlands; Maastricht University, Maastricht, Limburg, Netherlands; Maastricht University, Maastricht, Limburg, Netherlands; Maastricht University, Maastricht, Limburg, Netherlands; Maastricht University, Maastricht, Limburg, Netherlands; Maastricht University, Maastricht, Limburg, Netherlands

## Abstract

DAILy nurse is a complex intervention that was used in changing the breakfast routine for nursing home residents. It was expected that nursing home residents would be more engaged and would perform more activities independently; furthermore, it was expected that nursing staff behavior would change towards encouraging residents and taking over fewer activities. A quasi-experimental design with a pre- and posttest in four nursing homes was utilized. Participants were 104 residents and 134 staff. Video recordings were used to observe primary outcomes: engagement and independence in breakfast activities, and nursing staff activities. Results showed that staff succeeded in changing the breakfast routine dramatically. Departing from a standardized breakfast moment (with sandwiches fully prepared, no choice for residents) in a rather sterile environment (e.g., no ambiance, empty tables, almost no interactions), the routine was changed into a breakfast moment where tables were set with breadbaskets, different types of sandwich filling and pots of coffee and tea. This enabled residents to prepare their own sandwich and to be engaged in this activity, which led to interactions between residents; and residents and staff. Video recordings, and perceptions of nursing home staff, indicated statistically significant improvement of the daily lives of residents during the breakfast moment. However, no effect was found in nursing staff behavior. In conclusion, this study showed that applying DAILy NURSE, led to more engagement and independence of residents. It should be emphasized that this was feasible without increasing the number of staff or the allocation of extra budgets.

